# Results of targeted next-generation sequencing in children with cystic kidney diseases often change the clinical diagnosis

**DOI:** 10.1371/journal.pone.0235071

**Published:** 2020-06-23

**Authors:** Lena Obeidova, Tomas Seeman, Filip Fencl, Kveta Blahova, Jan Hojny, Veronika Elisakova, Jana Reiterova, Jitka Stekrova

**Affiliations:** 1 Institute of Biology and Medical Genetics, First Faculty of Medicine, Charles University and General University Hospital in Prague, Prague, Czech Republic; 2 Department of Pediatrics, 2^nd^ Faculty of Medicine, Charles University and Motol University Hospital, Prague, Czech Republic; 3 Institute of Pathology, First Faculty of Medicine, Charles University and General University Hospital in Prague, Prague, Czech Republic; 4 Department of Nephrology, First Faculty of Medicine, Charles University and General University Hospital in Prague, Prague, Czech Republic; All India Institute of Medical Sciences, Bhopal, INDIA

## Abstract

Cystic kidney diseases are a very heterogeneous group of chronic kidney diseases. The diagnosis is usually based on clinical and ultrasound characteristics and the final diagnosis is often difficult to be made. Next-generation sequencing (NGS) may help the clinicians to find the correct final diagnosis. The aim of our study was to test the diagnostic yield of NGS and its ability to improve the diagnosis precision in a heterogeneous group of children with cystic kidney diseases. Next-generation sequencing of genes responsible for the formation of cystic kidneys was performed in 31 unrelated patients with various clinically diagnosed cystic kidney diseases gathered at the Department of Pediatrics of Motol University Hospital in Prague between 2013 and 2018. The underlying pathogenic variants were detected in 71% of patients (n = 22), no or only one (in case of autosomal recessive inheritance) pathogenic variant was found in 29% of patients (n = 9). The result of NGS correlated with the clinical diagnosis made before the NGS in 55% of patients (n = 17), in the remaining 14 children (45%) the result of NGS revealed another type of cystic kidney disease that was suspected clinically before or did not find causal mutation in suspected genes. The most common unexpected findings were variants in nephronophthisis (NPHP) genes in children with clinically suspected autosomal recessive polycystic kidney disease (ARPKD, n = 4). Overall, 24 pathogenic or probably pathogenic variants were detected in the *PKHD1* gene, 8 variants in the *TMEM67* gene, 4 variants in the *PKD1* gene, 2 variants in the *HNF1B* gene and 2 variants in *BBS1* and *NPHP1* genes, respectively. NGS is a valuable tool in the diagnostics of various forms of cystic kidney diseases. Its results changed the clinically based diagnoses in 16% (n = 5) of the children.

## Introduction

Cystic kidney diseases (CKD) are a very heterogeneous group of chronic kidney diseases that in pediatric patients comprise mostly inherited kidney diseases, such as autosomal recessive polycystic kidney disease (ARPKD), nephronophthisis (NPHP), renal cysts and diabetes syndrome (RCAD) or autosomal dominant polycystic kidney disease (ADPKD) [1]. In contrary to children, in adults the most common cystic kidney disease are simple renal cysts or acquired renal cystic disease [1]. The diagnosis is usually based only on clinical and ultrasound characteristics and the final diagnosis is often difficult to be made.

In the last decade, next-generation sequencing becomes the method of choice, allowing simultaneous mutational analysis of panel of genes that can be especially useful in genetic diagnosis of young patients with unclear or doubtful phenotype, in etiologically complex syndromes, or in differential diagnosis of phenotypically overlapping syndromes. In very rare cases a combination of mutations in two genes (digenic disease), can be found in children with severe neonatal clinical phenotype [2]. Therefore, NGS may help the clinicians to make the correct final diagnosis in children with different types of chronic kidney diseases including cystic kidney diseases.

The aim of our study was to test the diagnostic yield of NGS and its ability to improve the diagnosis precision in a heterogeneous group of children with clinically diagnosed cystic kidney diseases.

## Materials and methods

### Patients and samples

Altogether, there were 31 patients with clinical diagnosis of different cystic kidney diseases–ARPKD, ADPKD, NPHP, RCAD syndrome and Bardet-Biedl syndrome. The group of patients was gathered at the Department of Pediatrics of Motol University Hospital in Prague between 2013 and 2018. The characterization of the group (age of disease manifestation, type of disease and sex distribution) is summarized in Table 1.

**Table 1:** Cohort characterization.

Clinical diagnosis		%	cases
	ARPKD	65	20
	ADPKD (VEO)	19	6
	RCAD syndrome	10	3
	NPHP	3	1
	Bardet-Biedl syndrome	3	1
**Age of disease manifestation**			
	Neonatal (prenatal)	48	15
	Infantile	26	8
	Childhood	26	8
**Sex distribution**			
	Male	58	18
	Female	42	13

ADPKD (VEO), autosomal dominant polycystic kidney disease with very early onset; ARPKD, autosomal recessive polycystic kidney disease; NPHP, nephronophthisis; RCAD, renal cysts and diabetes syndrome.

Clinical diagnosis of ARPKD was based on the fulfillment of established clinical criteria of ARPKD including 1) typical kidney involvement on ultrasound (enlarged hyperechogenic kidneys with bilateral poor corticomedullary differentiation), with or without 2) liver involvement (congenital hepatic fibrosis, ductal plate malformation) and 3) normal renal US of both parents consistent with autosomal-recessive inheritance [3].

Clinical diagnosis of ADPKD was based on the presence of one or more kidney cysts in one/both kidneys with or without positive family history of ADPKD and was mostly based on expert opinion as no consensus diagnostic criteria were stated for children under the age of 15. Retrospective evaluation shows that diagnosis of ADPKD in our patients followed the international consensus statement for radiological criteria for children and young people published in 2019 [4,5], where 1) findings of one or more kidney cysts in children under 15 years with a positive family history of ADPKD are highly suggestive of ADPKD and 2) multiple cysts with negative family history require clinical work-up for cystic kidney diseases.

The clinical diagnosis of nephronophthisis was based on the clinical finding of 1) polyuria/ polydipsia or 2) renal concentrating defect or 3) biopsy findings of chronic interstitial nephritis or US findings of normal sized hyperechogenic kidneys, all together with compatible autosomal recessive inheritance [6]. Nephronophthisis was also kept as a differential diagnosis in patients with kidney involvement and liver fibrosis. Nevertheless, in majority of cases diagnosis of ARPKD was left as a clinical diagnosis of first choice due to the rareness of NPHP, liver fibrosis in limited number of patients with NPHP or absence of eyes and central nervous system abnormalities in the patient.

Clinical diagnosis of renal cysts and diabetes syndrome (RCAD)–*HNF1B* nephropathy was mainly based on the presence of cystic kidney dysplasia with/without MODY and/or hypomagnesemia and/or pancreatic malformation, together with presence of other distinctive features described in detail in [7].

Clinical diagnosis of Bardet-Biedl syndrome was in our group of patients (patient n.25) based on the presence of cystic kidney disease, obesity, polydactyly and cognitive impairment as described among other features in [8].

Hypertension was defined as blood pressure ≥95^th^ percentile [9] and/or use of antihypertensive drugs at the time of NGS. Glomerular filtration rate was measured as estimated glomerular filtration rate according to Schwartz formula [10].

All children with severe cystic kidney disease or systematic disease with presence of cysts treated in the Department of Pediatrics of Motol University Hospital between 2013 and 2018 were included in our group of patients. Cases of typical ADPKD phenotype with mild manifestation and positive family history of the disease, as well as patients whose parents/legal guardians did not give informed consent, were excluded from the study.

Detailed clinical, laboratory and ultrasound information of patients are summarized in Table 2.

**Table 2:** Clinical data of patients.

Patient	Age at diagnosis	Parental renal ultrasound	Renal phenotype	Hepatic phenotype	HTN	eGFR (ml/min/ 1.73 m^2^)	Extra-renal/hepatic phenotype	Clinical diagnosis
1	Neonatal	Normal	Enlarged kidneys with multiple cysts	Hepatomegaly, periportal fibrosis (US)	Yes	25	Eyes (COMA)	ARPKD
2	Neonatal	Normal	Enlarged kidneys with multiple cysts	Normal	Yes	N/A (RRT since 12.9 years of age)	No	ADPKD–VEO
3	Infantile (6 months)	Normal	Enlarged kidneys with multiple cysts	Hepatomegaly, periportal fibrosis (US)	Yes	73	No	ARPKD
4	Neonatal	Normal	Enlarged kidneys with multiple cysts	Hepatomegaly, periportal fibrosis (US)	Yes	19	No	ARPKD
5	Neonatal (anhydramnios)	Normal	Enlarged kidneys with multiple cysts	Normal (US)	Yes	43	No	ARPKD
6	Childhood (15 months, hepatomegaly)	Normal	Enlarged kidneys with multiple cysts and calcifications	Hepatosplenomegaly, CHF (biopsy proven), esophageal varices	Yes	50	No	ARPKD
7	Neonatal (US screening)	Normal	Enlarged kidneys with multiple cysts	Hepatomegaly, periportal fibrosis (US)	Yes	54	No	ARPKD
8	Neonatal (polyhydramnios, RDS, disproportional growth)	Normal	Enlarged kidneys with increased echogenity	Normal	Yes	121	Enteral polyps	ARPKD
9	Infantile (6 months)	Mother ADPKD	Normal sized kidneys with multiple cysts	Hepatomegaly, multinodular hepatic cirrhosis (biopsy)	Yes	97	Insulin resistance, obesity	ADPKD
10	Infantile (1 year)	Normal	Small sized hyperechogenic kidneys with several cysts	Normal sized liver with periportal fibrosis (US)	Yes (nephrotic range proteinuria)	N/A (RRT since 12.0 years of age)	Aplasia of vagina and uterus, primary amenorrhea	ARPKD, MRKH syndrome
11	Neonatal (preterm delivery, 29^th^ gest. week, posthemorrhagic hydrocephalus)	Normal renal US (mother uterus duplex)	Normal sized hyperechogenic kidneys	Normal	Yes	82	Psychomotor retardation	RCAD syndrome
12	Infantile	Normal	Enlarged hyperechogenic kidneys with multiple cysts and calcifications	Normal	Yes	90	No	ARPKD
13	Neonatal (oligohydramnios)	Normal	Enlarged hyperechogenic kidneys	Normal	No	77	No	RCAD syndrome
14	Neonatal	Normal	Normal sized hyperechogenic kidneys	Hepatomegaly, periportal fibrosis (US)	Yes	N/A (RRT since 7.4 years of age)	Nystagmus	ARPKD
15	Infantile (3 months)	Normal	Enlarged hyperechogenic kidneys	Normal sized liver with periportal fibrosis (US)	Yes	60	No	ARPKD
16	Neonatal	Normal	Enlarged kidneys with multiple cysts	Hepato- splenomegaly, CHF (US)	Yes	N/A (RRT since 14.4 years of age)	No	ARPKD
17	Neonatal	Normal	Enlarged kidneys with multiple cysts	Hepatomegaly, periportal fibrosis with cysts (US)	Yes	21	No	ARPKD
18	Neonatal (anhydramnios)	Normal	Enlarged kidneys with multiple cysts	Hepatomegaly, periportal fibrosis with cysts (US)	Yes	N/A (RRT since 2.2 years of age)	Mild mental retardation	ARPKD
19	Neonatal	Normal	Enlarged kidneys with multiple cysts	Hepato- splenomegaly, CHF (US), esophageal varices	Yes	N/A (RRT since 18.5 years of age)	No	ARPKD
20	Infantile (4 months)	Normal	Hyperechogenic kidneys	Normal sized with irregular structure (US)	Yes	N/A (RRT since 4,9 years of age)	No	ARPKD
21	Neonatal	Normal	Enlarged kidneys with multiple cysts	Hepato- splenomegaly, CHF (US), esophageal varices	Yes	N/A (RRT since 17.7 years of age)	No	ARPKD
22	Neonatal	Father ADPKD	Enlarged kidneys with multiple cysts	Normal	Yes	87	No	ADPKD-VEO
23	Infantile (2 months)	Normal	Enlarged kidneys with multiple cysts	Hepatomegaly, periportal fibrosis (US)	Yes	95	No	ARPKD
24	Infantile (10 months)	Normal	Normal sized hyperechogenic kidneys	Hepatomegaly, periportal fibrosis (US)	Yes	N/A (RRT since 7.6 years of age)	No	ARPKD
25	Childhood (12 years)	Normal	Normal sized kidneys with cysts	Normal	No	57	Polydactyly, obesity, learning difficulties	BBS
26	Childhood (5 years)	Normal	Normal sized kidneys with cysts (disappearance of cysts in one kidney during follow-up)	Normal	No	98	Encapsulated abdominal hematoma (extirpated)	ADPKD
27	Childhood (2 years)	Normal	Normal sized kidneys with cysts	Normal	No	91	No	ADPKD
28	Childhood (5 years)	Normal	Normal sized kidneys with cysts	Normal	No	111	No	ARPKD
29	Childhood (8 years)	Normal	Normal sized hyperechogenic kidneys with cysts	Normal	No	18	Autism	NPHP
30	Childhood (8 years)	Normal	Normal sized kidneys with cortical cysts	Normal	No	127	Pancreatic cysts	RCAD syndrome
31	Childhood (3 years)	Normal	Normal sized kidneys with cortical cysts	Normal	Yes	116	No	ADPKD

ADPKD, autosomal dominant polycystic kidney disease; ARPKD, autosomal recessive polycystic kidney disease; BBS, Bardet-Biedl syndrome; CHF, congenital hepatic fibrosis; COMA, congenital oculomotor apraxia; eGFR, estimated glomerular filtration rate (at time of NGS) according to Schwartz formula; HTN, arterial hypertension; MRKH, Mayer-Rokitansky-Kuster-Hauser syndrome; NPHP, nephronophthisis; RCAD, renal cysts and diabetes syndrome; RDS, respiratory distress syndrome; RRT, renal replacement therapy; US, ultrasound; VEO, very early onset.

The study was approved by the Ethics Committee of General University Hospital in Prague and all patients/legal guardians were consulted by geneticist prior to molecular-genetic analysis and gave written informed consent for genetic testing.

### Molecular-genetic analysis

#### Next-generation sequencing

Two types of NGS library preparation were used within the project. For panel sequencing, target enrichment method with The SeqCap® EZ probe pool (Roche Sequencing Solutions) has been chosen. The full list of genes contained in the analyzed panel is in S1 Table (panel version 1) and S2 Table (panel version 2). The selection of genes contained in both panels reflected recommendations for ARPKD differential diagnosis presented in [3], systematic review of literature and demands of collaborating nephrologists and pediatricians. Both panels included genes associated with polycystic kidneys as well as other nephropathies to enable cost effective and rapid analysis of patients with different (and rare) diagnoses at once. During the years 2016 and 2017, the smaller panel (version 1) was analyzed in our patients. Starting the year 2018, the panel of genes has been extended (version 2) to incorporate more diagnoses and more causative genes. The sequencing library was prepared using Roche Sequencing Solutions following SeqCap EZ Library SR User’s Guide (version 5.1) with corresponding kits supplied by Roche (SeqCap Hybridization and Wash Kit, SeqCap Accessory Kit V2, SeqCap HE-Oligo Kit A and SeqCap HE-Oligo Kit B, and SeqCap Pure Capture Bead Kit), with preparation of samples for hybridization (including enzymatic fragmentation step) with KAPA HyperPlus Library Preparation Kit (technical data sheet n. KR1145—v2.15). Because of the presence of 6 pseudogenes with high homology to *PKD1*, library preparation from long-range PCR products was chosen for *PKD1* library preparation (primers and amplification conditions described by [11], PrimeSTAR® GXL DNA Polymerase (TaKaRa) was used for LR-PCR). The products were subsequently prepared for sequencing with Nextera XT DNA Library preparation kit (Illumina, Nextera XT DNA Library Preparation Guide Part # 15031942 Rev. E). Both libraries were sequenced on MiSeq sequencer (Illumina). The library preparation and sequencing of *PKD1* was done in cooperation with the Institute of Endocrinology in Prague. The quality metrics of panel sequencing are summarized in S3 Table. The sequencing of the *PKD1* gene was always followed by Sanger sequencing of regions with base coverage under 30x.

The bioinformatic analysis was provided by in-house bioinformatic pipeline. The analysis included detection of single-nucleotide polymorphisms (SNPs), small insertions and deletions and, in the case of panel sequencing, copy-number variants (CNV).

First, the quality of the FASTQ files created by MiSeq Reporter was evaluated using FastQC v0.11.5 (Babraham Institute). In the second step, cutting off the adapter (TruSeq3-PE-2) sequences (and PCR primers in case of *PKD1* sequencing) and quality check of the reads and their trimming was provided by Trimmomatic 0.36 [12]. The trimmed reads were again quality-checked with FastQC and mapped to reference genome with bwa.kit 0.7.12 (https://github.com/lh3/bwa/tree/master/bwakit). The hs38DH genome provided by bwa.kit was used. Mapped reads were cleaned up and sorted using samtools fixmate and samtools sort, respectively (samtools 1.6, http://www.htslib.org/doc/samtools.html). The PCR duplicates were then marked with picard MarkDuplicates 2.9.2-SNAPSHOT (Picard Toolkit by Broad Institute) and mapped reads indexed with samtools index (v1.6). For variant calling, freebayes v1.1.0-9-g09d4ecf was used [13]. The variant calling with freebayes was restricted to exons of analyzed genes with 5bp intron overlaps and included hard filtering of low-quality variants. The variants were annotated with SnpEff 4.3r [14] and SnpSift 4.3r. [15] compiling information from prediction programs, conservation scores, allele frequencies etc. included in databases such as dbSNP (https://www.ncbi.nlm.nih.gov/snp/) and dbNSFP 3.2a (https://sites.google.com/site/jpopgen/dbNSFP).

The variant prioritization was composed of automatic filtration (point 1 and 2) followed by evaluation by molecular geneticist in cooperation with nephrologist (points 3–5):

1. The variant causes change on protein level (i.e. nonsynonymous sequence changes in exons) or the variant is in the intronic region in the donor (+1, +2, +4, +5) or acceptor (-1, -2) splite site.
2. The variant has a population frequency (of European descend) lower than 0.1. 1000 Genome Project EUR population and Exome Aggregation Consortium (ExAC) was used for the assessment.
3. Evaluation of the variant based on *in silico* prediction by programs: SIFT [16], PolyPhen-2 [17], LRT [18], MutationTaster (http://www.mutationtaster.org/), MutationAssessor [19], FATHMM [20], PROVEAN [21], CADD [22], ensemble scores MetaSVM [23], and conservation scores.
4. The consultation of variant with VarSome search engine [24] containing data retrieved from archive of clinically relevant interpretations of variants ClinVar [25], protein database Uniprot [26] etc., and reports of variant pathogenicity using automated classifier evaluating variants according to the ACMG guidelines [27]. Consultation of variant with Human Gene Mutation Database Professional (HGMD) and specialized mutation databases: Mutation Database Autosomal Recessive Polycystic Kidney Disease (ARPKD/PKHD1, http://www.humgen.rwth-aachen.de/) and Autosomal Dominant Polycystic Kidney Disease Mutation Database: PKDB (https://pkdb.mayo.edu/).
5. The consultation with attending nephrologist regarding the possible pathogenic effect of detected variants with uncertain significance on patient phenotype.
6. If available, analysis of variant segregation in family members. In case of recessive inheritance, analysis of variation in proband’s parents to determine *trans* localization in the patient.

The copy-number variant calling was done using CNVkit 0.8.6.dev0 [28] in all samples analyzed with panel sequencing.

Patients 1–24 had had mutational analysis of the *PKHD1* gene done within our previous project. These patients were either clinically suspected to have ARPKD or had neonatal/infantile onset of the disease. The sequencing method, data analysis and variant classification used in this project were in detail described previously [29]. The definitive results (two causal mutation in the *PKHD1* gene) of patients 3–5 and 7 were already published and discussed in [29]. The patients with definitive genetic diagnosis provided by previous sequencing of *PKHD1* gene alone (e.g. two probable causal mutations found within the *PKHD1* gene located in trans) have not been analyzed by panel or *PKD1* sequencing (patients 3–5, 7, 15–18, 21, 23). Patient 24 was analyzed in commercial laboratory. The complete list of patients and their respective genetic analysis are summarized in S4 Table.

#### MLPA

MLPA (Multiplex Ligation-dependent Probe Amplification) analysis was used for detection of large genome rearrangements. MLPA of *PKHD1* and *HNF1B* followed the genetic analysis of the *PKHD1* gene in patients in whom no/one mutation in *PKHD1* was found. The kits used for MLPA analysis were SALSA MLPA P341 PKHD1 mix 1, P342-PKHD1 mix 2 and SALSA MLPA P241 MODY Mix 1 (MRC Holland). The generated data were analyzed by Coffalyser.Net (MRC Holland).

The MLPA analysis was also applied in patient 29 to confirm the complete deletion of both alleles of *NPHP1* gene found by panel sequencing. The kit used for the analysis was SALSA MLPA P387 NPHP1.

## Results

The group of 31 patients formed by patients from Motol University Hospital gathered between years 2013 and 2018 was analyzed within our project. The underlying pathogenic variants were detected in 71% of patients (n = 22), no or only one (in case of suspected disease with autosomal recessive inheritance) pathogenic variant was found in 29% of patients (n = 9) (Table 3). The result of NGS correlated with the clinical diagnosis made before the NGS analysis in 55% of patients (n = 17), in the remaining 14 children (45%) the result of NGS did not confirm the clinical diagnosis or diagnosed another type of cystic kidney disease than suspected clinically. In 20 patients, clinical diagnosis of ARPKD was suggested. The sequencing of *PKHD1* confirmed the diagnosis in 10 (50%) of them. In the other 10 patients, the sequencing of *PKD1* and panel of genes yielded the diagnosis in additional 5 of them (nephronophthisis in 4 patients, 1 patient with ADPKD-VEO). Four children harbored only one pathogenic mutation in the recessive *PKHD1* gene, and the final diagnosis remained unknown.

**Table 3:** Summary of variants identified in the patients.

Patient	Gene	DNA sequence change	Predicted change on protein level	dbSNP ID	Prediction of pathogenity	Inheritance	Clinical diagnosis	Genetic diagnosis	Agreement of clinical and genetic diagnosis
1	***TMEM67***	**NM_153704.6:c.1843T>C**	**p.(Cys615Arg)**	**rs201893408**	**Likely Pathogenic**	**P**	ARPKD	NPHP11	No
	***TMEM67***	**NM_153704.6:c.1843T>C**	**p.(Cys615Arg)**	**rs201893408**	**Likely Pathogenic**	**M**			
2	*PKD1*	*NM_001009944*.*3*:*c*.*6965C>T*	*p*.*(Thr2322Met)*	*rs564570407*	*--*	*--*	ADPKD–VEO	Unknown	No
	*NEK8*	*NM_178170*.*3*:*c*.*133C>T*	*p*.*(Arg45Trp)*	*rs1567759130*	*--*	*--*			
	*COQ8B*	*NM_024876*.*4*:*c*.*767C>A*	*p*.*(Ala256Glu)*	*rs201827222*	*--*	*--*			
3	***PKHD1*** ^***a***^	**NM_138694.4:c.5895dupA**	**p.(Leu1966ThrfsTer4)**	**rs746838237**	**Pathogenic**	**P**	ARPKD	ARPKD	Yes
	***PKHD1*** ^***a***^	**NM_138694.4:c.8114delG**	**p.(Gly2705ValfsTer11)**	**rs774050795**	**Pathogenic**	**M**			
4	***PKHD1*** ^***a***^	**NM_138694.4:c.107C>T**	**p.(Thr36Met)**	**rs137852944**	**Likely Pathogenic**	**M**	ARPKD	ARPKD	Yes
	***PKHD1*** ^***a***^	**NM_138694.4:c.7561_7568delGCAGCAAT**	**p.(Ala2521PhefsTer60)**	**N/A**	**Pathogenic**	**--**			
5	***PKHD1*** ^***a***^	**NM_138694.4:c.107C>T**	**p.(Thr36Met)**	**rs137852944**	**Likely Pathogenic**	**M**	ARPKD	ARPKD	Yes
	***PKHD1*** ^***a***^	**NM_138694.4:c.10658T>C**	**p.(Ile3553Thr)**	**rs137852948**		**P**			
6	***PKHD1*** ^***a***^	**NM_138694.4:c.8114delG**	**p.(Gly2705ValfsTer11)**	**rs774050795**	**Pathogenic**	**P**	ARPKD	Unknown	No
7	***PKHD1*** ^***a***^	**NM_138694.4:c.4870C>T**	**p.(Arg1624Trp)**	**rs200391019**	**Uncertain Significance**	**P**	ARPKD	ARPKD	Yes
	***PKHD1*** ^***a***^	**NM_138694.4:c.5323C>T**	**p.(Arg1775Ter)**	**rs770522674**	**Pathogenic**	**M**			
8	*KIF7*	*NM_198525*.*3*:*c*.*2227C>T*	*p*.*(Gln743Ter)*	*N/A*	*--*	*--*	ARPKD	Bilateral blastema nephroblastoma	No
	*GLIS2*	*NM_032575*.*2*:*c*.*737G>A*	*p*.*(Arg246His)*	*rs770824489*	*--*	*--*			
9	***PKD1***	**NM_001009944.3:c.5653dupG**	**p.(Glu1885GlyfsTer105)**	**N/A**	**Pathogenic**	**M**	ADPKD	ADPKD	Yes
10	*------*						ARPKD, MRKH syndrome	Unknown	No
11	***HNF1B***	**whole gene deletion**				**M**	RCAD syndrome	RCAD syndrome	Yes
12	***PKHD1***	**NM_138694.4:c.920T>C**	**p.(Ile307Thr)**	**rs1288017883**	**Likely Pathogenic**	**--**	ARPKD	Unknown	No
	*ACTN4*	*NM_004924*.*6*:*c*.*1279G>A*	*p*.*(Ala427Thr)*	*rs201128110*	*--*	*--*			
13	***HNF1B***	**NM_000458.4:c.523A>T**	**p.(Lys175Ter)**	**N/A**	**Pathogenic**	**--**	RCAD syndrome	RCAD syndrome	Yes
14	***TMEM67***	**NM_153704.6:c.1843T>C**	**p.(Cys615Arg)**	**rs201893408**	**Likely Pathogenic**	**In trans**	ARPKD	NPHP11	No
	***TMEM67***	**NM_153704.6:c.1815_1831del**	**p.(Gln605HisfsTer17)**	**N/A**	**Pathogenic**	**In trans**			
15	***PKHD1***	**NM_138694.4:c.107C>T**	**p.(Thr36Met)**	**rs137852944**	**Likely Pathogenic**	**P**	ARPKD	ARPKD	Yes
	***PKHD1***	**NM_138694.4:c.8114delG**	**p.(Gly2705ValfsTer11)**	**rs774050795**	**Pathogenic**	**--**			
16	***PKHD1***	**NM_138694.4:c.2725C>T**	**p.(Arg909Ter)**	**rs727504089**	**Pathogenic**	**--**	ARPKD	ARPKD	Yes
	***PKHD1***	**NM_138694.4:c.8870T>C**	**p.(Ile2957Thr)**	**rs760222236**	**Likely Pathogenic**	**M**			
17	***PKHD1***	**NM_138694.4:c.4403T>C**	**p.(Leu1468Pro)**	**rs140331370**	**Likely Pathogenic**	**P**	ARPKD	ARPKD	Yes
	***PKHD1***	**NM_138694.4:c.8870T>C**	**p.(Ile2957Thr)**	**rs760222236**	**Likely Pathogenic**	**M**			
18	***PKHD1***	**NM_138694.4:c.5323C>T**	**p.(Arg1775Ter)**	**rs770522674**	**Pathogenic**	**M**	ARPKD	ARPKD	Yes
	***PKHD1***	**NM_138694.4:c.5060T>C**	**p.(Ile1687Thr)**	**rs794727566**	**Pathogenic**	**P**			
19	***PKHD1***	**NM_138694.4:c.107C>T**	**p.(Thr36Met)**	**rs137852944**	**Likely Pathogenic**	**M**	ARPKD	Unknown	No
	*ZNF423*	*NM_015069*.*4*:*c*.*1144T>C*	*p*.*(Ser322Pro)*	*rs142835239*	*Likely Benign*	*--*			
20	***TMEM67***	**NM_153704.6:c.1843T>C**	**p.(Cys615Arg)**	**rs201893408**	**Likely Pathogenic**	**P**	ARPKD	NPHP11	No
	***TMEM67***	**NM_153704.6:c.1843T>C**	**p.(Cys615Arg)**	**rs201893408**	**Likely Pathogenic**	**M**			
21	***PKHD1***	**NM_138694.4:c.107C>T**	**p.(Thr36Met)**	**rs137852944**	**Likely Pathogenic**	**P**	ARPKD	ARPKD	Yes
	***PKHD1***	**NM_138694.4:c.9719G>A**	**p.(Arg3240Gln)**	**rs146649803**	**Likely Pathogenic**	**M**			
22	***PKD1***	**NM_001009944.3:c.12442G>T**	**p.(Glu4148Ter)**	**N/A**	**Pathogenic**	**De novo**	ADPKD-VEO	ADPKD	Yes
	*PKD1*	*NM_001009944*.*3*:*c*.*11084A>G*	*p*.*(His3695Arg)*	*N/A*	*--*	*M*			
23	***PKHD1***	**NM_138694.4:c.664A>G**	**p.(Ile222Val)**	**rs369925690**	**Likely Pathogenic**	**P**	ARPKD	ARPKD	Yes
	***PKHD1***	**NM_138694.4:c.100G>A**	**p.(Gly34Arg)**	**N/A**	**Uncertain Significance**	**M**			
24	***TMEM67***	**NM_153704.6:c.1843T>C**	**p.(Cys615Arg)**	**rs201893408**	**Likely Pathogenic**	**--**	ARPKD	NPHP11	No
	***TMEM67***	**NM_153704.6:c.1843T>C**	**p.(Cys615Arg)**	**rs201893408**	**Likely Pathogenic**	**--**			
25	***BBS1***	**NM_024649.5:c.46_47delAG**	**p.(Ser16GlnfsTer2)**	**rs1291184039**	**Pathogenic**	**P**	BBS	BBS	Yes
	***BBS1***	**NM_024649.5:c.1660_1661delAG**	**p.(Leu555GlnfsTer2)**	**rs1209299063**	**Pathogenic**	**M**			
26	***PKHD1***	**NM_138694.4:c.8870T>C**	**p.(Ile2957Thr)**	**rs760222236**	**Likely Pathogenic**	**P**	ADPKD	Unknown	No
	*TMEM237*	*NM_001044385*.*3*:*c*.*52C>T*	*p*.*(Arg18Ter)*	*rs199469707*	*--*	*M*			
27	***PKD1***	**NM_001009944.3:c.10619-2A>G**		**N/A**	**Pathogenic**	**de novo**	ADPKD	ADPKD	Yes
28	***PKD1***	**NM_001009944.3:c.6090delC**	**p.(Val2031TrpfsTer85)**	**N/A**	**Pathogenic**	**de novo**	ARPKD	ADPKD	No
29	***NPHP1***	**Deletion of whole gene**				**--**	NPHP	NPHP1	Yes
	***NPHP1***	**Deletion of whole gene**				**--**			
30	*AGXT*	*NM_000030*.*3*:*c*.*33dupC*	*p*.*(Lys12GlnfsTer156)*	*rs180177201*	*--*	*--*	RCAD syndrome	Unknown	No
	*RET*	*NM_020975*.*6*:*c*.*2372A>T*	*p*.*(Tyr791Phe)*	*rs77724903*	*--*	*--*			
31	*FREM1*	*NM_144966*.*5*:*c*.*3359A>T*	*p*.*(Gln1120Leu)*	*rs143844459*	*--*	*--*	ADPKD	Unknown	No
	*FREM1*	*NM_144966*.*5*:*c*.*3331C>T*	*p*.*(His1111Tyr)*	*rs200339767*	*--*	*--*			

ADPKD, autosomal dominant polycystic kidney disease; ARPKD, autosomal recessive polycystic kidney disease; BBS, Bardet-Biedl syndrome; M, maternal; MRKH, Mayer-Rokitansky-Kuster-Hauser syndrome; NPHP, nephronophthisis; P, paternal; RCAD, renal cysts and diabetes syndrome; VEO, very early onset.

Pathogenicity predictions was generated by VarSome [24] according to the ACMG guidelines [27]. Additional variants without clear significance on phenotype are in italics.

^a^ The results were already published in [29]

In patients without a yield by sequencing of *PKHD1* and those with childhood onset, without a clinical suspicion of ARPKD, a panel sequencing and sequencing of the *PKD1* gene yielded the diagnosis in 57% patients (12 of 21). The most common unexpected findings were changes in nephronophthisis (NPHP) genes in children with clinically suspected ARPKD (n = 4). The most common incorrect clinical diagnosis was ARPKD (n = 10) with four patients having genetically proven NPHP, one ADPKD de novo and further five patients having no definitive genetic diagnosis.

Overall, 24 pathogenic or probably pathogenic SNP variants were detected in the *PKHD1* gene (14 patients), 8 variants in the *TMEM67* gene (4 patients), 4 variants in the *PKD1* gene (4 patients), 1 variant in *HNF1B* (1 patient) and 2 variants in *BBS1* (1 patient). Moreover, whole gene deletion was detected in heterozygous (*HNF1B* gene) and homozygous (*NPHP1* gene) state in two patients.

The molecular genetic data of patients are summarized in Table 3.

## Discussion

Sequencing analysis performed in 31 pediatric patients with suspected cystic kidney diseases detected causal variant in 71% (n = 22) of patients. This roughly correlates with results presented by Bullich et al. [30] where targeted sequencing of 140 genes associated with formation of cystic or glomerular nephropathies yielded the diagnosis in 81% of prenatally and 71% postnatally presented pediatric patients with CKD. In two patients, whole gene deletion of *HFN1B* and *NPHP1* was detected. In both genes, whole gene deletions are the most common variants described in literature [31,32].

NGS confirmed the clinical diagnosis in 55% (n = 17) patients and did not confirm or changed the clinical diagnosis in 45% (n = 14) of them. The most common clinical diagnosis that had to be changed thanks to the NGS results was ARPKD. Four children (patients 1, 14, 20 and 24) with a clinical diagnosis of ARPKD was diagnosed as having genetically NPHP11 and one patient (28) de novo ADPKD. The clinical and ultrasound picture of ARPKD and NPHP can be similar making the differential diagnosis difficult mainly in children with non-neonatal manifestation in ARPKD and in absence of extra-renal/hepatic manifestation such as eyes or central nervous system abnormalities present in NPHP. The less severe NPHP phenotype in our patients was caused by the fact that all children carried the allele p.Cys615Arg (three in homozygous and one in heterozygous state with another *TMEM67* variant), which was already described in 2009 by Otto et al. [33] as hypomorphic allele associated with phenotype of NPHP with hepatic fibrosis and no brain anomaly.

In 9 patients, the genetic diagnosis remained unknown after NGS analysis. Some of these patients manifested with untypical clinical picture of the disease (patient n. 2, 26 and 31) or had another clinical diagnosis discovered during follow-up (patient n.8 - bilateral blastema nephroblastoma, patient n. 10—Mayer-Rokitansky-Kuster-Hauser syndrome). In three children (15%) with clinically diagnosed ARPKD only one pathogenic variant in the *PKHD1* gene was found. These findings are in concordance with literature, as single heterozygous *PKHD1* variant was detected in 36% of 164 ARPKD patients in study by Bergmann et al. [34]. Even though by definition the heterozygous carriers should not show any clinical disease manifestation, study by Gunay-Aygun et al. [35] suggests that individuals who are heterozygous for *PKHD1* mutations have an increased risk of polycystic liver disease and mild PKD. Nevertheless, in this study patients showed very mild renal and/or hepatic phenotype with normal renal function which is in strict contrast with our patients who had severe renal and/or hepatic phenotype.

No or partial genetic findings in some patients could be caused by several reasons:

Mutations in other ciliopathic genes which were not in our NGS ciliopathy panel (e.g. *DZIP1L* [36], *CEP83* [37], or many other genes that are gradually described in literature as causing ciliopathies) could cause the phenotype of cystic kidney disease in unresolved patients.

Furthermore, the causal pathogenic variant could be located in sequences that were not (or poorly) covered by genetic analysis, e.g. regulatory regions in deep intronic sequences, untranslated regions of genes, first exons of genes or even promotor regions, that are especially important for genes coding transcription factors whose function strongly relates to expression level. Also, the causing variant could have been filtered out as variant of unknown significance (VUS) as its function is yet unknown or could have been missed by bioinformatic processing.

Moreover, a combination of heterozygous mutations in two different ciliopathic recessive (or dominant) genes could theoretically cause a severe cystic kidney disease phenotype as an oligogenic inheritance where variants in different genes modify final phenotype (“mutational load” hypothesis) was already described in ciliopathies (reviewed in [38]), as well as in severe forms of PKD [2]. Although, we did find some suspected combinations of variants in some of our patients (e.g. 22, 26) we could not confirm the possible effect of these variants on final phenotype of a child.

Another possibility for the discrepancy between clinical and genetic diagnosis is that the cystic kidney disease in a given child is non-inherited (acquired, mainly solitary cysts) which is however very rare in pediatric patients especially if manifested in neonatal/infant age.

Extra-renal manifestations, such as eye or CNS abnormalities were the best clues for the correct clinical diagnosis of patients with non-ADPKD cystic kidney diseases [1]. Nystagmus, COMA, polydactyly, autism or mental retardation lead to suspicion of NPHP or BBS in several of our patients (Table 2). Therefore, the extra-renal findings are very important in making the correct diagnosis and they should be sought in all children with cystic kidney disease.

The strengths of our study are a very well clinical and ultrasound characterization of the patients, the long-term follow-up and the high number of ciliopathy genes in our panel. The genetic diagnosis consisted of several methods of sequencing and CNV detection and thus provided reliable results. We are aware of the limitations of our study—the low number of patients, single-center study, lack of some non-ciliopathy genes in our panel and lack of whole-exome sequencing in the genetically unresolved patients.

In conclusions, our study demonstrated that the next-generation sequencing method facilitates molecular analysis of different types of cystic kidney diseases and enables precise genetic diagnosis. Its results changed the clinical diagnosis in number of the children. The correct final genetic diagnoses enable better management of children with different forms of cystic kidney diseases and provide efficient genetic counseling.

## Supporting information

S1 Table Full list of genes contained in panel version number 1. (PDF)

S2 Table Full list of genes contained in panel version number 2. (PDF)

S3 Table Quality metrics of panel sequencing. (PDF)

S4 Table Summary of molecular-genetic analysis provided for each patient within our projects. +, yes; -, no.
(PDF)
