# Supplementary material for: Results of targeted next-generation sequencing in children with cystic kidney diseases often change the clinical diagnosis
Source: PLoS One. 2020 Jun 23;15(6):e0235071. doi: 10.1371/journal.pone.0235071 (PMC7310724; doi:10.1371/journal.pone.0235071)
Supplement: S1 Table — (PDF) [file pone.0235071.s001.pdf]

ACTN4	NM_004924
AHI1	NM_017651
ALMS1	NM_015120
ANKS6	NM_173551
ANLN	NM_018685
APOL1	NM_003661
ARHGDIA	NM_001185077
ARL13B	NM_182896
ARL6	NM_032146
B9D1	NM_015681
B9D2	NM_030578
BBIP1	NM_001195304
BBS1	NM_024649
BBS10	NM_024685
BBS12	NM_152618
BBS2	NM_031885
BBS4	NM_033028
BBS5	NM_152384
BBS7	NM_176824
BBS9	NM_198428
BICC1	NM_001080512
BVES	NM_007073
C3	NM_000064
CC2D2A	NM_001080522
CD2AP	NM_012120
CD46	NM_002389
CEP164	NM_014956
CEP290	NM_025114
CEP41	NM_018718
CFB	NM_001710
CFI	NM_000204
CLCNKB	NM_000085
COL4A3	NM_000091
COL4A4	NM_000092
COL4A5	NM_000495
COQ8B	NM_024876
CPLANE1	NM_023073
CRB2	NM_173689
DGKE	NM_003647
DYNC2H1	NM_001080463
EMP2	NM_001424
EYA1	NM_000503
GDNF	NM_000514
GFRA1	NM_005264
GLIS2	NM_032575
HNF1B	NM_000458
IFT140	NM_014714
IFT172	NM_015662

IFT27	NM_001177701
IFT80	NM_020800
INF2	NM_022489
INVS	NM_014425
IQCB1	NM_001023570
KCNJ1	NM_000220
KIF14	NM_014875
KIF7	NM_198525
LAMC1	NM_002292
LMX1B	NM_002316
LZTFL1	NM_020347
MKKS	NM_018848
MKS1	NM_017777
MUC1	NM_002456
MYH9	NM_002473
MYO1E	NM_004998
NAT8	NM_000169
NEK8	NM_178170
NPHP1	NM_000272
NPHP3	NM_153240
NPHP4	NM_015102
NPHS1	NM_004646
NPHS2	NM_014625
OFD1	NM_003611
PAX2	NM_003990
PEX1	NM_000466
PEX10	NM_153818
PEX11B	NM_003846
PEX13	NM_002618
PEX14	NM_004565
PEX16	NM_057174
PEX19	NM_002857
PEX2	NM_000318
PEX26	NM_017929
PEX3	NM_003630
PEX5	NM_001300789
PEX6	NM_000287
PKD1	NM_001009944
PKD2	NM_000297
PKHD1	NM_138694
PLCE1	NM_016341
PMPCA	NM_019892
PTPRO	NM_030667
REN	NM_000537
RET	NM_020975
RPGRIP1L	NM_015272
SDCCAG8	NM_006642
SLC12A1	NM_000338

SLC12A3	NM_000339
TCTN1	NM_001082538
TCTN2	NM_024809
TCTN3	NM_015631
THBD	NM_000361
TMEM138	NM_001044385
TMEM216	NM_016499
TMEM231	NM_001077416
TMEM237	NM_152388
TMEM67	NM_153704
TRIM32	NM_012210
TRPC6	NM_004621
TSC1	NM_000368
TSC2	NM_000548
TTC21B	NM_024753
TTC8	NM_144596
UMOD	NM_003361
VHL	NM_000551
WDPCP	NM_015910
WDR19	NM_025132
WT1	NM_024426
ZNF423	NM_015069
