# Supplementary material for: Results of targeted next-generation sequencing in children with cystic kidney diseases often change the clinical diagnosis
Source: PLoS One. 2020 Jun 23;15(6):e0235071. doi: 10.1371/journal.pone.0235071 (PMC7310724; doi:10.1371/journal.pone.0235071)
Supplement: S2 Table — (PDF) [file pone.0235071.s002.pdf]

ACE	NM_000789.3
ACTN4	NM_001322033.1
AGT	NM_000029.3
AGTR1	NM_000685.4
AGXT	NM_000030.2
AHI1	NM_017651.4
ANKS6	NM_173551.4
ANLN	NM_018685.4
APOL1	NM_145343.2
AQP2	NM_000486.5
ARHGDIA	NM_004309.5
ARL13B	NM_182896.2
ARL6	NM_032146.5
ATP6V0A4	NM_020632.2
ATP6V1B1	NM_001692.3
AVP	NM_000490.4
AVPR2	NM_000054.4
B9D1	NM_015681.4
B9D2	NM_030578.3
BBIP1	NM_001195305.1
BBS1	NM_024649.4
BBS10	NM_024685.3
BBS12	NM_152618.2
BBS2	NM_031885.3
BBS4	NM_033028.4
BBS5	NM_152384.2
BBS7	NM_018190.3
BBS9	NM_001033604.1
BICC1	NM_001080512.2
BMP4	NM_001202.5
BSND	NM_057176.2
C3	NM_000064.3
CASR	NM_000388.3
CC2D2A	NM_001080522.2
CD2AP	NM_012120.2
CD46	NM_172361.2
CEP104	NM_014704.3
CEP164	NM_014956.4
CEP41	NM_018718.2
CFB	NM_001710.5
CFH	NM_000186.3
CFHR1	NM_002113.2
CFHR3	NM_021023.5
CFHR5	NM_030787.3
CFI	NM_000204.4
CLCNKA	NM_001042704.1
CLCNKB	NM_000085.4
CLDN16	NM_006580.3

CLDN19	NM_148960.2
COL4A1	NM_001303110.1
COL4A2	NM_001846.3
COL4A3	NM_000091.4
COL4A4	NM_000092.4
COL4A5	NM_000495.4
COL4A6	NM_001287758.1
COQ2	NM_015697.8
COQ6	NM_182476.2
COQ8B	NM_024876.3
CPLANE1	NM_023073.3
CRB2	NM_173689.6
DGKE	NM_003647.2
DSTYK	NM_015375.2
DYNC2H1	NM_001080463.1
DZIP1L	NM_173543.2
EMP2	NM_001424.5
EYA1	NM_000503.5
FGF20	NM_019851.2
FN1	NM_212476.2
FRAS1	NM_001166133.1
FREM1	NM_144966.5
GANAB	NM_198334.2
GATA3	NM_001002295.1
GLIS2	NM_032575.2
GRHPR	NM_012203.1
GRIP1	NM_001178074.1
HNF1B	NM_000458.3
HOGA1	NM_138413.3
CHD1L	NM_004284.5
IFT80	NM_020800.2
INF2	NM_022489.3
INVS	NM_014425.4
IQCB1	NM_001023570.3
ITGA8	NM_003638.2
KCNJ1	NM_000220.4
KIF7	NM_198525.2
LAMC1	NM_002292.3
LMX1B	NM_001174146.1
LRP5	NM_002335.3
LZTFL1	NM_020347.3
MAGED2	NM_014599.5
MKKS	NM_018848.3
MKS1	NM_017777.3
MUC1	NM_002456.5
MYH9	NM_002473.5
MYO1E	NM_004998.3
NEK8	NM_178170.2

NPHP1	NM_000272.3
NPHP3	NM_153240.4
NPHP4	NM_001291593.1
NPHS1	NM_004646.3
NPHS2	NM_001297575.1
NUP107	NM_020401.3
NUP205	NM_015135.2
NUP93	NM_014669.4
OFD1	NM_003611.2
PAX2	NM_000278.4
PDSS2	NM_020381.3
PKHD1	NM_138694.3
PLCE1	NM_016341.3
PMPCA	NM_019892.5
PRKCSH	NM_001001329.2
PTPRO	NM_002848.3
REN	NM_000537.3
RET	NM_020975.5
ROBO2	NM_002942.4
RPGRIP1L	NM_015272.4
SALL1	NM_002968.2
SDCCAG8	NM_006642.4
SEC63	NM_007214.4
SIX1	NM_005982.3
SIX2	NM_016932.4
SIX5	NM_175875.4
SLC12A1	NM_000338.2
SLC12A3	NM_000339.2
SLC34A1	NM_003052.4
SLC3A1	NM_000341.3
SLC4A1	NM_000342.3
SLC7A9	NM_001126335.1
SLC9A3R1	NM_004252.4
SOX17	NM_022454.3
TCTN1	NM_024549.5
TCTN2	NM_024809.4
TCTN3	NM_015631.5
THBD	NM_000361.2
TMEM138	NM_016464.4
TMEM216	NM_016499.5
TMEM231	NM_001077416.2
TMEM237	NM_152388.3
TMEM67	NM_153704.5
TRAF3IP1	NM_015650.3
TRIM32	NM_012210.3
TRPC6	NM_004621.5
TSC1	NM_000368.4
TSC2	NM_000548.4

TTC21B	NM_024753.4
TTC8	NM_144596.3
UMOD	NM_001008389.2
UPK3A	NM_006953.3
VHL	NM_000551.3
WDPCP	NM_015910.6
WDR19	NM_001317924.1
WT1	NM_000378.5
ZNF423	NM_015069.4
