# Supplementary material for: Results of targeted next-generation sequencing in children with cystic kidney diseases often change the clinical diagnosis
Source: PLoS One. 2020 Jun 23;15(6):e0235071. doi: 10.1371/journal.pone.0235071 (PMC7310724; doi:10.1371/journal.pone.0235071)
Supplement: S3 Table — (PDF) [file pone.0235071.s003.pdf]

Sample	PCT_TARGET_BASES_10X	PCT_TARGET_BASES_20X	PCT_TARGET_BASES_30X	PCT_TARGET_BASES_40X	PCT_TARGET_BASES_50X	PCT_TARGET_BASES_100X	TOTAL_READS	ON_BAIT_BASES	NEAR_BAIT_BASES	OFF_BAIT_BASES	PCT_OFF_BAIT	MEAN_TARGET_COVERAGE	MEDIAN_TARGET_COVERAGE
**Panel version number 1**													
01_88512	0.986043	0.978481	0.9701	0.958565	0.944627	0.820353	1791240.0	132895028.0	54997701.0	84772126.0	0.310902	144.675831	149.0
02_88412	0.985922	0.97937	0.971933	0.96267	0.949991	0.855488	1905015.0	141074139.0	60271005.0	91411002.0	0.312243	156.364834	159.0
06_147912	0.988088	0.983652	0.979353	0.974693	0.968848	0.918775	2535499.0	185700742.0	82164332.0	126260535.0	0.320356	207.255224	200.0
08_134013	0.988503	0.982872	0.977716	0.971466	0.964273	0.905573	2393991.0	180887459.0	77387212.0	113321323.0	0.304958	201.044251	200.0
10_94312	0.986981	0.981383	0.975561	0.967741	0.958907	0.88728	1896584.0	140070229.0	64034564.0	91006114.0	0.308379	159.118378	163.0
11_117812	0.992705	0.988887	0.985343	0.981909	0.978184	0.950359	2520432.0	202928151.0	94922889.0	91521738.0	0.235049	225.060637	200.0
12_88612	0.986937	0.980511	0.973055	0.964843	0.954897	0.895106	2598645.0	196132526.0	79496802.0	125093968.0	0.31217	208.961153	200.0
13_136012	0.987768	0.981948	0.975015	0.963965	0.947668	0.747774	1646876.0	119023841.0	52293062.0	84653969.0	0.330717	133.371499	130.0
14_88712	0.990414	0.986183	0.981305	0.976	0.970548	0.905066	1872557.0	148696153.0	72898243.0	67977058.0	0.234751	169.474523	177.0
19_76512	0.988393	0.982267	0.975197	0.965891	0.955753	0.873233	1991811.0	146411797.0	60185082.0	94031557.0	0.312783	157.408126	161.0
20_88912	0.988872	0.982649	0.975577	0.966524	0.957096	0.885695	2371582.0	176692330.0	67780691.0	116036641.0	0.321868	182.534007	187.0
22_71615	0.986802	0.978346	0.967917	0.954358	0.938691	0.733043	1413352.0	104840147.0	47332470.0	66121255.0	0.3029	120.060344	122.0
26_104016	0.990963	0.987902	0.983949	0.979601	0.974498	0.931516	2375817.0	188210434.0	84003180.0	90638088.0	0.249794	203.8827	200.0
27_51515	0.986567	0.978736	0.96999	0.959694	0.948419	0.838385	1588230.0	121085374.0	64484687.0	60231916.0	0.245042	142.234584	149.0
28_44916	0.992172	0.986556	0.981534	0.974999	0.966772	0.865032	1765541.0	117467994.0	79869698.0	71068134.0	0.264779	146.983224	152.0
**Panel version number 2**													
30_123818	0.992151	0.988947	0.985378	0.980751	0.971852	0.830827	2607217.0	70247121.0	59969010.0	64323253.0	0.330644	124.654797	128.0
31_173718	0.997611	0.995822	0.993471	0.990895	0.98763	0.937422	3703952.0	99068138.0	81487737.0	97653848.0	0.351008	175.432504	180.0
32_166818	0.995761	0.992987	0.989622	0.985459	0.979855	0.883982	2914182.0	73578073.0	62328625.0	82576834.0	0.377954	130.322397	132.0

PCT_TARGET_BASES_10X	The fraction of all target bases achieving 10X or greater coverage.
PCT_TARGET_BASES_20X	The fraction of all target bases achieving 20X or greater coverage.
PCT_TARGET_BASES_30X	The fraction of all target bases achieving 30X or greater coverage.
PCT_TARGET_BASES_40X	The fraction of all target bases achieving 40X or greater coverage.
PCT_TARGET_BASES_50X	The fraction of all target bases achieving 50X or greater coverage.
PCT_TARGET_BASES_100X	The fraction of all target bases achieving 100X or greater coverage.
TOTAL_READS	The total number of reads in the SAM or BAM file examined.

ON_BAIT_BASES	The number of PF_BASES_ALIGNED that are mapped to the baited regions of the genome.
NEAR_BAIT_BASES	The number of PF_BASES_ALIGNED that are mapped to within a fixed interval containing a baited region, but not within the baited section per se.
OFF_BAIT_BASES	The number of PF_BASES_ALIGNED that are mapped away from any baited region.
PCT_OFF_BAIT	The fraction of PF_BASES_ALIGNED that are mapped away from any baited region, OFF_BAIT_BASES/PF_BASES_ALIGNED.
MEAN_TARGET_COVERAGE	The mean coverage of a target region.
MEAN_TARGET_COVERAGE	The mean coverage of a target region.
