# Supplementary material for: Results of targeted next-generation sequencing in children with cystic kidney diseases often change the clinical diagnosis
Source: PLoS One. 2020 Jun 23;15(6):e0235071. doi: 10.1371/journal.pone.0235071 (PMC7310724; doi:10.1371/journal.pone.0235071)
Supplement: S4 Table — +, yes; -, no. (PDF) [file pone.0235071.s004.pdf]

| Patient | PKHD1 sequencing | Panel sequencing (1): Panel v.1 (2): Panel v.2 | MLPA PKHD1 | MLPA HNF1B | PKD1 sequencing |
|---|---|---|---|---|---|
| 1/88512 | + | + (1) | + | + | + |
| 2/88412 | + | + (1) | + | + | + |
| 3/89313 | + | - | - | - | - |
| 4/135812 | + | - | - | - | - |
| 5/117712 | + | - | - | - | - |
| 6/147912 | + | + (1) | + | + | + |
| 7/135912 | + | - | - | - | - |
| 8/134013 | + | + (1) | + | + | + |
| 9/137113 | + | - | - | - | + |
| 10/94312 | + | + (1) | + | + | + |
| 11/117812 | + | + (1) | + | + | + |
| 12/88612 | + | + (1) | + | + | + |
| 13/136012 | + | + (1) | + | + | - |
| 14/88712 | + | + (1) | + | + | + |
| 15/88812 | + | - | - | - | - |
| 16/86512 | + | - | - | - | - |
| 17/86312 | + | - | - | - | - |
| 18/88312 | + | - | - | - | - |
| 19/76512 | + | + (1) | + | + | + |
| 20/88912 | + | + (1) | + | + | + |
| 21/86412 | + | - | - | - | - |
| 22/71615 | + | + (1) | + | + | + |
| 23/37815 | + | - | - | - | - |
| 24/09416 | + | Commercial lab | + | - | - |
| 25/104016 | - | + (1) | - | - | + |
| 26/51515 | - | + (1) | - | - | + |
| 27/44916 | - | + (1) | - | - | + |
| 28/118418 | - | - | - | - | + |
| 29/123818 | - | + (2) | - | - | - |
| 30/173718 | - | + (2) | - | - | - |
| 31/166818 | - | + (2) | - | - | + |
